# Volcanic related methylmercury poisoning as the possible driver of the end-Devonian Mass Extinction

**DOI:** 10.1038/s41598-020-64104-2

**Published:** 2020-04-30

**Authors:** Michał Rakociński, Leszek Marynowski, Agnieszka Pisarzowska, Jacek Bełdowski, Grzegorz Siedlewicz, Michał Zatoń, Maria Cristina Perri, Claudia Spalletta, Hans Peter Schönlaub

**Affiliations:** 1 Faculty of Natural Sciences, University of Silesia in Katowice, Będzińska 60, 41- 200 Sosnowiec, Poland; 2 Institute of Oceanology, Polish Academy of Sciences, Powstańców Warszawy 55, 81-712 Sopot, Poland; 3 Department of Biological, Geological and Environmental Sciences, University of Bologna, via Zamboni 67, 40126 Bologna, Italy; 4 Austrian Academy of Sciences, Commission for Geosciences, 2, Dr. Ignaz Seipel-Platz, Vienna, 1010 Austria

**Keywords:** Geochemistry, Palaeontology, Palaeoceanography

## Abstract

The end-Devonian global Hangenberg event (359 Ma) is among the most devastating mass extinction events in Earth’s history, albeit not one of the “Big Five”. This extinction is linked to worldwide anoxia caused by global climatic changes. These changes could have been driven by astronomical forcing and volcanic cataclysm, but ultimate causes of the extinction still remain unclear. Here we report anomalously high mercury (Hg) concentration in marine deposits encompassing the Hangenberg event from Italy and Austria (Carnic Alps). The Hangenberg event recorded in the sections investigated can be here interpreted as caused by extensive volcanic activity of large igneous provinces (LIPs), arc volcanism and/or hydrothermal activity. Our results (very large Hg anomalies) imply volcanism as a most possible cause of the Hangenberg event, similar to other first order mass extinctions during the Phanerozoic. For the first time we show that apart from anoxia, proximate kill mechanism of aquatic life during the event could have been methylmercury formed by biomethylation of a volcanically derived, huge concentration of inorganic Hg supplied to the ocean. Methylmercury as a much more toxic Hg form, potentially could have had a devastating impact on end-Devonian biodiversity, causing the extinction of many pelagic species.

## Introduction

The end-Devonian was a time of significant changes in the global climate and biosphere, including the biodiversity crisis known as the Hangenberg event^1,2^. This event occurred ca. 13.5 ± 0.5 Ma after the Frasnian-Famennian mass extinction, and was linked with globally widespread deposition of the anoxic Hangenberg Black Shale^1–4^. The Hangenberg extinction (with 50% marine genera loss) significantly affected the pelagic realm, especially ammonoids^5^, conodonts^6^, many vertebrates^3^ and benthic reef biotas, such as trilobites and ostracods^1^, and had an ecological impact similar to the end-Ordovician mass extinction^7^. Moreover, a drastic reduction of phytoplankton diversity is also observed at the Devonian/Carboniferous (D/C) boundary^8^. Deposition of the Hangenberg black shale was a short-term event that lasted between ~50 and 190 kyr^9^, while the extended crisis interval encompassed a time span of one to several hundred kyr^1^. The postulated factors responsible for this global event, such as high productivity and anoxia, a calcification crisis caused by ocean acidification, perturbation of the global carbon cycle, glacio-eustatic sea-level changes driven by orbital forcing, volcanic and hydrothermal activity, and evolution of land plants, are still vividly discussed^1^. In fact, extensive volcanism has been implicated in all ‘Big Five’ mass extinctions and other biotic crises in the Phanerozoic^10–14^, including the Hangenberg crisis^9,15^. As the main source of mercury (Hg) in the geological past was volcanic and submarine hydrothermal activity^16,17^, and Hg anomalies in the sedimentary record have recently been used as a proxy for volcanic activity in relation to global events and paleoenvironmental perturbations^18–21^, also for the D/C boundary from different paleogeographical domains^22–25^.

Here we report very large, anomalous Hg spikes in two marine D/C successions of the Carnic Alps, supporting volcanism^11,12^ as the driving mechanism (ultimate cause) of the Hangenberg event. Furthermore, we also detected methylmercury (MeHg), a strong neurotoxin that bioaccumulates in the food chain^26–29^, in sedimentary rocks for the first time. Thus, we claim that volcanic-driven methylmercury poisoning in otherwise anoxic seas could be an another proximate (direct) kill mechanism of the end-Devonian Hangenberg extinction.

## Study Areas

**Figure 1:**
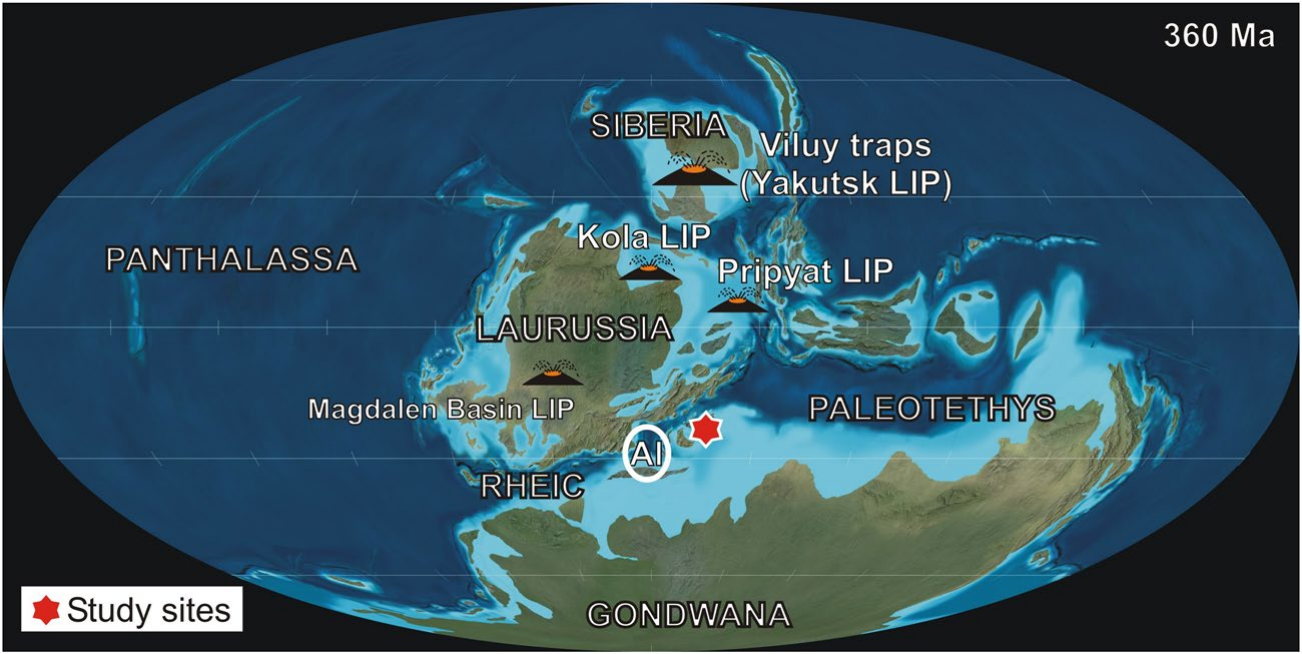
Late Devonian (360 Ma) paleogeographic map (after^65^) showing the studied localities and the location of prominent areas of Late Devonian magmatism and associated volcanism^51,54,66,67^, as well as (Al) giant mercury deposits reactivated by Variscan magmatic and tectonic activity in Almadén (Spain)^68^.

**Figure 2:**
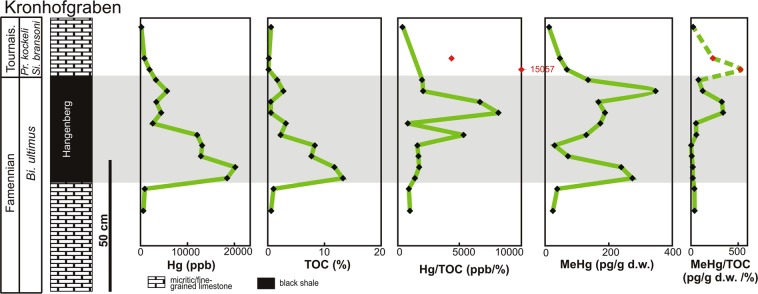
Reference Devonian/Carboniferous (D/C) section in Kronhofgraben (Carnic Alps, Austria) showing Hg enrichment and MeHg levels associated with the Hangenberg event. Red points indicate the sample with low total organic carbon (TOC) values (≤0.2%). Biostratigraphy based on^32^. Mass extinctions level are marked in grey. Abbreviation: *Bi*. – *Bispathodus*, *Pr*. – *Protognathodus* and *Si*. – *Siphonodella,* Tournais – Tournaisian.

We examined two successions of deep-water, pelagic sedimentary rocks, encompassing the uppermost Devonian and D/C boundary intervals (Fig. 1): Kronhofgraben (Austria) and Plan di Zermula A (Italy) in the Carnic Alps^30^. The Kronhofgraben and Plan di Zermula A sections consist of organic-rich Hangenberg black shale (HBS) and micritic limestone (Fig. 2).

The Kronhofgraben section (N 46°36′00.0″, E 13° 02′ 02.0″) in the central Carnic Alps (Austria) is situated in a gorge of the Aßnitz Creek, ca. 7 km east of Plöckenpass and ca. 1 km northwest of the Kronhof Törl pass at the Austrian–Italian border. The D/C boundary beds crop out in the eastern side of the Kronhofgraben gorge at an altitude of 1390 m^31^. The Plan di Zermula A section (N 46°34*'* 31.0*"*, E 13° 06*'* 41.0*"*) in the southern Carnic Alps (Italy) appears on the western slope of the Mount Zermula massif, along the road from Paularo to Stua di Ramaz. Grey limestones and black shales represent the studied interval in both sections. The Hangenberg Black Shale (HBS) horizon assigned to the upper part of the *Bispathodus ultimus* Zone (=Middle-Upper *praesulcata* zones) in Kronhofgraben (40 cm thick) and in Plan di Zermula A (15 cm thick) is underlain by cephalopod limestones of the lower part of the Bispathodus ultimus Zone (=Upper *expansa*- Lower *praesulcata* zones)^32^. The first carbonate bed above the HBS belongs to the *sulcata* Zone (=*Protognathodus kockeli* Zone p.p.)^30,32–35^.

The D/C boundary in both sections is situated directly above the Hangenberg Black Shale^30,31^,^33^. The D/C boundary may be somewhat problematic and needs redefinition (caused by problems with discrimination of *Siphonodella sulcata* from its supposed ancestor *Siphonodella praesulcata*^35^). The new criterion for definition of the base of the Carboniferous System proposed by the Working Group on the boundary is: identification of the base of the *Pr. kockeli* Zone, beginning of radiation and top of major regression (top of HSS) and end of mass extinction^32^. In the limestone overlying the HBS, the conodonts of the species *Protognathodus kockeli*^32^ was found in both sections. Therefore, the position of the D/C boundary did not changed in comparison to previous studies^30,31^,^33^.

The D/C boundary successions in the Plan de Zermula A and Kronhofgraben were deposited in deeper paleoenvironment^31^. In the late Devonian, Carnic Alps represented the northern tips of Gondwana and belonged to the Gondwana-derived Bosnian–Noric Terrane accreted to the intra-Alpine Mediterranean terrane during the Carboniferous^36^. The investigated rocks outcropped in the Carnic Alps reflected strong thermal alteration (CAI) ranging from 4.5 to 5 in the Plan di Zermula A and Kronhofgraben, respectively^31,37^.

## Results

**Figure 3:**
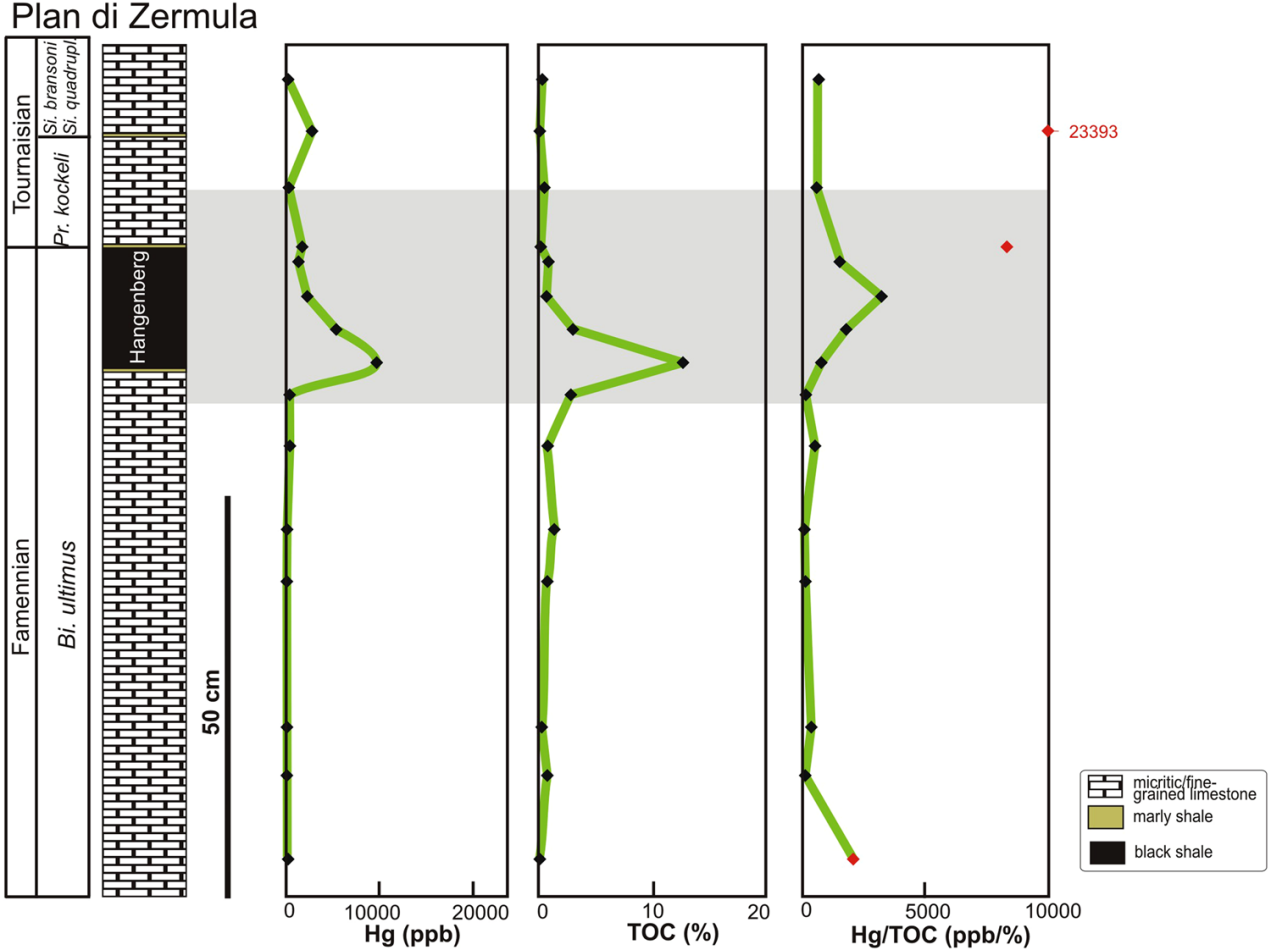
Reference Devonian/Carboniferous (D/C) section in Plan di Zermula A (Carnic Alps, Italy) showing Hg enrichment associated with the Hangenberg event. Red points indicate samples with low total organic carbon (TOC) values (≤0.2%). Biostratigraphy based on^32^. Mass extinctions level are marked in grey. Abbreviation: *Bi*. – *Bispathodus*, *Pr*. – *Protognathodus* and *Si*. – *Siphonodella*.

The HBS intervals in the sections investigated display extremely high Hg values, with maxima of 20216 and 9758 ppb in Kronhofgraben (Fig. 2; Table 1) and Plan di Zermula A (Fig. 3; Table 2), respectively. The HBS from the Plan di Zermula A section contains Hg anomalies that are ~13–100 times higher than the ~100 ppb background, whereas in the Kronhofgraben section the anomalies are ~12–84 times higher than the background values.

**Table 1:** MeHg (pg/g d.w.), Hg (ppb), TOC (%), TS (%), Mo (ppm) and Al_2_O_3_ (%) content and Hg/TOC (ppb/%), Hg/TS (ppb/%) and MeHg/TOC (pg/g d.w./%) in Kronhofgraben section, Carnic Alps (Austria). The horizons with low TOC content are marked by asterisks.

Sample	MeHg (pg/g d.w.)	Hg (ppb)	TOC (%)	TS (%)	Mo (ppm)	Al_2_O_3_ (%)	Hg/TS (ppb/%)	Hg/TOC (ppb/%)	MeHg/TOC (pg/g d.w./%)
KR 12 C	13.0	221	0.57	4.94	0.4	0.61	44.74	388	23
KR 12B	46.7	864	0.2*	3.77	1.3	2.1	229.18	4320*	234*
KR 12 A	68.6	1957	0.13*	3.55	0.8	1.96	551.27	15057*	528*
KR 11	135.2	3288	1.67	2.05	24.3	19.63	1603.90	1969	81
KR 10	347.6	5695	2.78	0.65	25.4	15.63	8761.54	2049	125
KR 9	167.9	3377	0.51	4.10	3.8	14.47	823.66	6622	329
KR 8	188.6	4453	0.55	5.70	43.4	12.88	781.23	8097	343
KR 7	173.9	2625	3.22	1.79	5.8	15.38	1466.48	815	54
KR 6	129.9	12107	2.28	6.32	31.8	18.65	1915.66	5310	57
KR 5	29.9	13150	8.26	5.49	96.1	13.17	2395.26	1592	4
KR 4	72.3	12894	7.72	2.15	70.2	11.49	5997.21	1670	9
KR 3	239.8	20216	11.78	1.06	103.6	13.75	19071.70	1716	20
KR 2	275.0	18466	13.28	0.46	100.4	12.87	40143.48	1391	21
KR 1	38.5	914	1.03	5.78	2.1	1.07	158.13	888	37
KR 0	24.5	611	0.62	2.49	4.1	0.93	245.38	985	40

**Table 2:** Hg (ppb), TOC (%),TS (%), Mo (ppm) and Al_2_O_3_ (%) content and Hg/TOC (ppb/%) and Hg/TS (ppb/%) in Plan di Zermula A section, Carnic Alps (Italy). The horizons with low TOC content are marked by asterisks.

Sample	Hg (ppb)	TOC (%)	TS (%)	Mo (ppm)	Al_2_O_3_ (%)	Hg/TS (ppb/%)	Hg/TOC (ppb/%)
PZ 4	223	0.33	3.03	0.8	1.27	73.60	677
PZ 3	2807	0.12*	0.72	0.8	22.11	3898.61	23393*
PZ 2	305	0.52	2.36	0.8	1.23	129.24	587
PZ 1	1745	0.2*	0.12*	2.3	15.91	14541.67*	8723*
PZ 0D	1345	0.87	0.81	9.1	10.58	1660.49	1546
PZ 0C	2289	0.7	0.31	3.2	15.85	7383.87	3269
PZ 0B	5406	2.99	0.69	6.8	15	7834.78	1808
PZ 0A	9758	12.53	0.38	72.5	12.54	25678.95	779
PZ 02	391	2.81	4.04	1.6	0.85	96.78	139
PZ 03B	419	0.8	5.93	0.4	0.56	70.66	524
PZ 03A	116	1.37	3.34	1.4	0.89	34.73	85
PZ 04	93	0.77	5.49	0.8	1.08	16.94	121
PZ 05	102	0.28	0.08	0.4	24.68	1275.00	365
PZ 06	87	0.76	0.99	0.2	1.23	87.88	114
PZ 07	232	0.11*	0.02*	0.2	22.91	11600.00*	2107*

Interestingly, significant concentrations of methylmercury were found in the whole Kronhofgraben section, where MeHg is in the range 13–348 pg/g d.w. (Fig. 2). Additionally, we found 55 pg/g d.w. of MeHg in Novchomok section in Uzbekistan and 72.72 pg/g d.w. of MeHg sampled from the uppermost Devonian part of the Woodford Shale from the Arbuckle Anticline in the Oklahoma, USA. Traces of MeHg were also found in the HBS interval at Kowala Quarry, Poland (20.66 pg/g d.w. of MeHg).

In comparison to MeHg levels found in modern sediments (reaching from1000 to 700000 pg/g d.w. in polluted basin^38^), those detected in sedimentary rocks studied, are relatively low. However, the original amounts of MeHg in the investigated sediments would have been higher but impoverished during diagenesis (see below for details). The Hg enrichments are observed in organic-rich Hangenberg equivalent intervals such as Kronhofgraben (from 0.51 to 13.28% TOC) and Plan di Zermula A (from 0.7 to 12.53% TOC). The values of the Hg/TOC ratio in the HBS at Kronhofgraben range from 815 to 8096.5 (ppb/%), while the background samples show a range from 387.5 to 985 (ppb/%). In Plan di Zermula A, the values of Hg/TOC ratios in the HBS range from 779 to 3269 (ppb/%) and are higher than those from the background samples (ranging from 84.5 to 676.8 ppb/%Hg/TOC).

## Discussion and Conclusions

**Figure 4:**
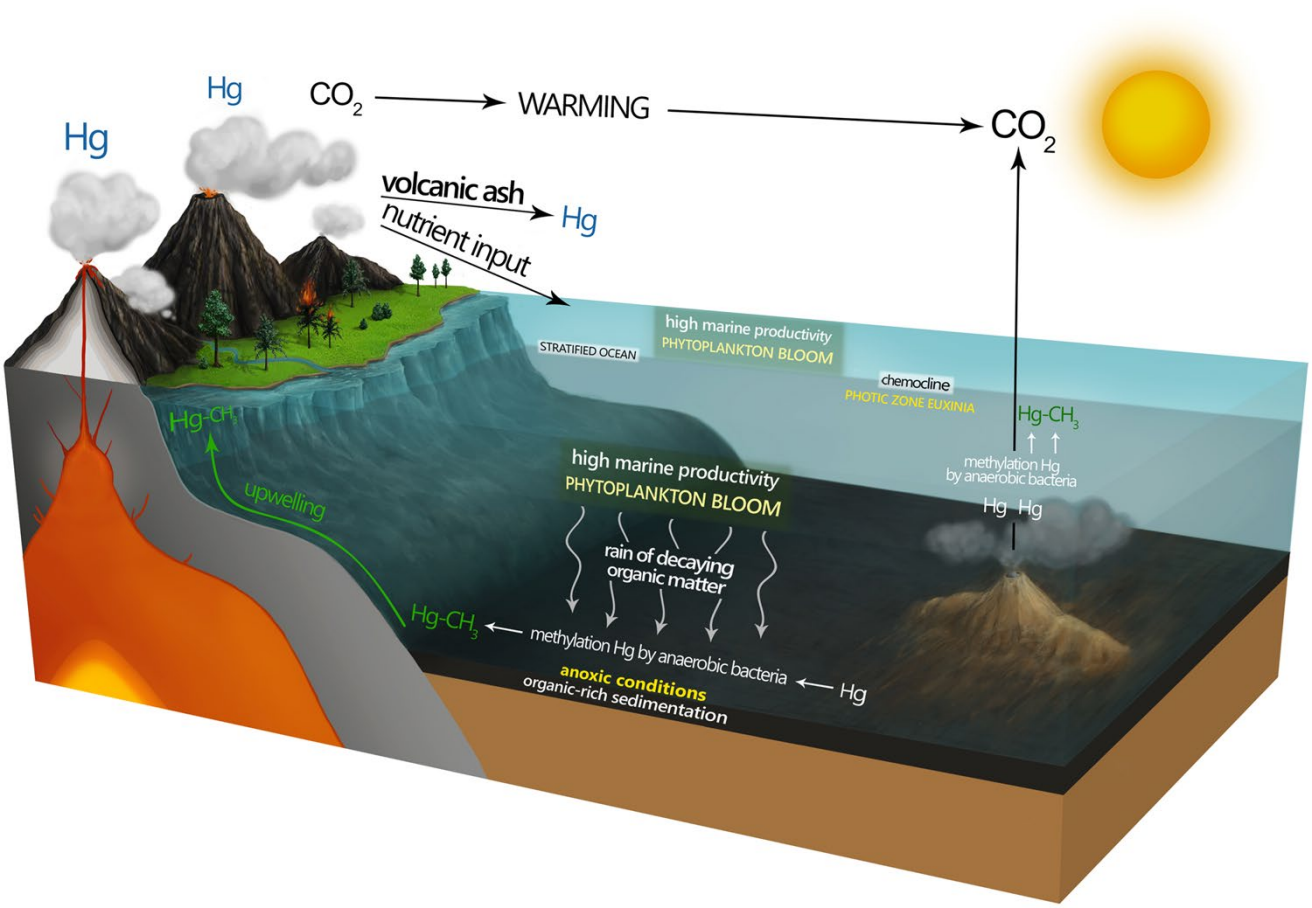
Schematic model of deposition, Hg sources and Hg methylation during the Hangenberg event.

Volcanic and hydrothermal activities are considered to be the main sources of elevated Hg in sedimentary rocks^16,17,39–41^. Besides Hg delivery to the atmosphere by volcanic activity, other processes can produce Hg spikes in the sedimentary record, including widespread wildfires, terrestrial input, magmatic emplacement or thermogenic processes related to bolide impact^19,39,40,42–44^. Additionally, some authors suggested that Hg enrichments can be sulfide-hosted in euxinic facies, and high Hg spikes not necessary would be connected with volcanic activity^45^. However, in such a case, the Hg enrichments would be well-correlated with total sulphur (TS), which is not observed in our sections. Although extensive wildfires on land were confirmed during the Hangenberg event, based on the co-occurrence of charcoal and high concentrations of polycyclic aromatic hydrocarbons in sedimentary rocks^2,46,47^, these, however, could have also been induced by volcanism, as evidenced by the co-occurrence of charcoals and ash layers^2,46^. No conclusive evidence for bolide impact at the D/C boundary has been detected thus far^1^. In fact, at the D/C boundary, volcanic activity has frequently been documented, mainly on the basis of the presence of ash layers below, above and within the HBS (e.g. in the Holy Cross Mountains, Iberian Pyrite Belt (SW Iberia) and Rhenish Massif^2,9,15,48^), mercury spikes (e.g.^14^,^23^–^25^), as well as the presence of abnormal or strongly altered spores (tetrads), which could reflect the mutagenic effect of regional acidification caused by explosive volcanism^47,49,50^. The most plausible sources of very large amounts of Hg during the end-Devonian interval are the massive Magdalen silicic large igneous province (LIP)^12^ and the Siberian (Yakutsk–Viluy) and/or the Kola–Dnieper LIPs^12,51^; however, the interval also overlaps with formation of the Almaden Hg deposit (last mineralization pulse episodes^52^), which constitutes one of the largest geochemical anomalies on Earth and coincided with the first phase of the Variscan orogeny^53^, as considered for the Hangenberg crisis^15^. According to current knowledge, three LIPs encompass the Late Devonian interval (380–360 Ma): Yakutsk-Viluy (Siberia; continental type with area of 0.8 Mkm^2^), Kola-Dnieper (Baltica; continental type with area of 3 Mkm^2^) and Magdalen (Laurussia; continental-silic type)^12^. Moreover, we cannot exclude other additional Hg sources, for instance connected with explosive eruptions which could overlap with LIPs activity, (see e.g. Magdalen Basin^54^). Mercury has a strong affinity to organic matter and to a minor extent can also be associated with sulfides and clay minerals; therefore, Hg is normalized to total organic carbon (TOC) content^19^. Importantly, the Hg spikes in our sections are also evident when normalized to TOC content, which can be interpreted as an effect of increased input of Hg to the basins independently of the potential influence of reducing depositional conditions. The Hg vs. Al_2_O_3_ correlation in the investigated successions is very weak (R^2^ = 0.25 in Kronhofgraben and R^2^ = 0.09 in Plan di Zermula A), indicating no correlation of Hg with the clay fraction. However, Hg exhibits a good correlation with Mo in the all sections (R^2^ = 0.88 in Kronhofgraben and R^2^ = 0.76 in Plan di Zermula A). This could indicate that some Hg was associated with sulfides as a result of its intensified precipitation in a sulfide-rich (euxinic) water column^2,42,45^. In the sections investigated, Hg vs. TS correlation is very weak (R^2^ = 0.10 in Kronhofgraben and R^2^ = 0.16 in Plan di Zermula A) which does not confirm sulfides as host of Hg. However, the Hg vs. TOC correlation in the D/C boundary at Novchomok section is very low^22^, which confirm that Hg enrichments are facies independent and thus are indicative of volcanic activity during this time. For the Kronhofgraben and Plan di Zermula A sections this correlation is good (R^2^ = 0.86 and 0.75 respectively), suggesting possible different sources of this element. However, as already emphasized, there are a number of lines of evidence for volcanic and hydrothermal activities, as well as widespread wildfires, during this time^2,15^, allowing for a firm statement that increased Hg input to the basins was connected with diverse volcanic activities and related combustion of biomass on land. Moreover, the Hangenberg event took place during an interglacial period^1^; therefore, some Hg could have originated from permafrost melting^55^, but even if this process had taken place, Hg would have previously accumulated in the permafrost as a result of volcanic or pyrogenic processes. To summarize, based on all the available data, we can state that the main sources of Hg were volcanism and related hydrothermal activities (Fig. 4; see also^23,24^). In fact, volcanic processes are main sources of Hg in atmosphere^39^.

The organic form of mercury (CH_3_Hg^+^) is a strong neurotoxin that is bioconcentrated in aquatic food chains and is able to cross the blood–brain barrier; thus, this form of Hg is much more toxic to living organisms than inorganic Hg^26,28^. In modern environments, methylmercury (MeHg) is generated predominantly by anaerobic microorganisms, such as sulfate-reducing bacteria (e.g., *Geobacter sulfurreducens*)^56^. Despite widespread Hg pollution, annual emissions of Hg have recently been higher from natural sources than anthropogenic ones, constituting as much as 70% of all Hg emissions^57^. However, the concentrations of Hg detected in all the end-Devonian sections are surprisingly high, similar to the present-day mercury concentrations found in highly polluted basins, e.g., some parts of the Baltic Sea^38^. The Hg concentrations of up to 20000 ppb in Kronhofgraben and 1000–10000 ppb in the Plan di Zermula A, and Hg spikes determined in Germany^22^, south Vietnam^23^, Czech Republic and south China^24^ sections suggest, that global Hg concentrations were highly elevated during the Hangenberg event. This finding implies that, during favorable sedimentary conditions, very high concentrations of MeHg can be produced on the global scale. In the investigated samples we measured relatively minor amounts of MeHg (Table 1) in comparison with the MeHg levels in modern sediments. In polluted basins, concentrations of MeHg vary from 1000 to 700000 pg/g d.w. of MeHg^38^ and are much higher relative to total MeHg concentration from our sections. However, the original amounts of MeHg in the investigated sediments would have been higher, assuming large enrichment of total Hg in anomalous samples. It is very probable that MeHg could have been demethylated during diagenesis as a result of the common diagenetic process of demethylation, which is influenced by temperature^58^. Because of the strong thermal alteration of the investigated rocks (conodont alteration index = 4.5–5 in Carnic Alps^31,37^) the occurrence of demethylation seems to be very likely.

Therefore, regardless of the Hg source, its high level in the end-Devonian water column, subsequent trapping in sediment and biomethylation to the more toxic MeHg form by anaerobic bacteria, would have had an additional devastating impact on aquatic life during the Hangenberg event. This can be produced under conditions of extended anoxia/euxinia during this time^2,59^ and the occurrence of rich sulfate-reducing bacteria communities^60^ which can change Hg to its methyl form. Additionally, blooms of green algal phototrophs (prasinophytes)^8^ during black shale events would have contributed, mostly indirectly, to MeHg production^61^. However, indisputable evidence for bacterial Hg methylation is the occurrence of notable concentrations of MeHg in the sediments investigated and the similarities in the distributions of Hg and MeHg in the Kronhofgraben section (Fig. 2).

Observation of modern marine environments has confirmed that MeHg is highly toxic to animals at higher trophic levels (such as fish, birds and mammals)^26,28^. In this light it seems to be evident that severe extinction of marine and nonmarine fish and tetrapods^3^, as well as pelagic conodont animals^1^, during the Hangenberg event may also have resulted from MeHg poisoning that could have affected different aquatic habitats. Although the effect of MeHg on benthic invertebrates is regarded as minimal^62^, these organisms were significantly affected by concomitant, globally widespread anoxia. Such anoxia asphyxiation–methylmercury poisoning may have also been kill mechanisms in other mass extinctions, but this should be tested by searching for traces of methylmercury in other sedimentary rocks.

## Methods

A total of 30 samples were analyzed for Hg abundances, using atomic absorption spectrometry (AAS) analyzer Milestone DMA-80 Direct Mercury (detection limit = 0.2 ppb). An Eltra CS-500 IR-analyzer was used for total organic carbon (TOC) determination [for more detail methodology see^22^]. In addition, Hg as well as Mo and Al contents were analyzed using ICP-MS at the Bureau Veritas Acme Labs Canada Ltd. The results of Hg concentrations measured by AAS and ICP-MS are comparable^22^. Mercury enrichments are typically normalized to TOC^42^. For some samples, Hg/TOC ratios were deemed unreliable, due to very low TOC content (<0.2 TOC%; these samples are marked in red on Figs. 2–3). In these cases even low Hg concentration may generate very high, artificial Hg/TOC spikes^63^. Additionally, we examined 19 samples in terms of methylmercury (MeHg) occurrence (Kronhofgraben, n = 15; Novchomok, Uzbekistan n = 1; Arbuckle Anticline USA n = 1; Kowala, Poland n = 1 and one blank sample form Morocco La10 – Lahmida, which consist of only trace amounts of total Hg < 7 ppb). MeHg was determined using Automated Methylmercury System MERX-M (Brooks Rand, USA) following EPA Method 1630. Samples were extracted according to the modified method of De Wild *et al*.^64^. In brief, five grams of grinded rock was weighted in a 30 ml Teflon centrifuge tube. For each tube 5 ml of extraction buffer (180 g KBr, 50 ml concentrated H_2_SO_4_, 0.2 g NH_2_OH ∙ HCl dilute to 1 liter), 1 ml of 1 M CuSO_4_ and 10 ml of CH_2_Cl_2_ were added. Tubes were left at room temperature in darkness for 1 h, and afterwards they were mixed on a vortex mixer for 1 h in darkness. Then, the samples were centrifuged at 3000 rpm for 30 min. From each sample 5 ml of CH_2_Cl_2_ phase was added into a back-extraction vial containing 25 ml of reaction grade water. The back-extraction vials were placed in a heating block (45 °C) and attached to N_2_ lines (flow at 100 mL per minute). After the extraction, 5 ml of the aqueous sample was added to 20 ml of reaction grade water in a glass reaction vial. The pH was adjusted to between 4 and 5 with the addition of 300 µl of 2 M acetate buffer. The last step was an addition of 50 µl of 1% NaBEt_3_. Samples were analyzed using Automated Methylmercury System MERX-M (Brooks Rand, USA) following EPA Method 1630.

The Method Detection Limit (MDL) value was determined based on a series of measurement results of blank samples spiked with extracts obtained from the reference material ERMCC580 (Estuarine sediment MeHg = 75 ng/g d.w. ± 4 ng/g d.w.). Nine determinations were performed for extracts diluted to the expected MDL value - 1 pg. The MDL was calculated as 3x Standard Deviation (SD). Method Quantification Limit (MQL) was calculated as 3x MDL. The MDL value was estimated to be 0.7 pg what corresponds to MeHg concentration in sediments samples of an even 3.5 pg/g d.w. (calculated from the sample weight that was taken for extraction and including sample moisture - 2 g d.w.). The MQL value was calculated as MQL = 3xMDL and equalling 2.2 pg what corresponds to a MeHg concentration of 11.0 pg/g d.w. (assuming the mass of the sample - 2 g of d.w.).

The mercury-poor sample (Hg = 6.78 ppb) from the Lahmida section used here as reference blank contain MeHg below detection limit (2.73 pg/g d.w.).
